# Percutaneous Hepatic Perfusion (PHP) with Melphalan in Liver-Dominant Metastatic Uveal Melanoma: The German Experience

**DOI:** 10.3390/cancers14010118

**Published:** 2021-12-27

**Authors:** Cornelia L. A. Dewald, Mia-Maria Warnke, Roland Brüning, Martin A. Schneider, Peter Wohlmuth, Jan B. Hinrichs, Anna Saborowski, Arndt Vogel, Frank K. Wacker

**Affiliations:** 1 Institute for Diagnostic and Interventional Radiology, Hannover Medical School, 30623 Hannover, Germany; hinrichs.jan@mh-hannover.de (J.B.H.); wacker.frank@mh-hannover.de (F.K.W.); 2 Department of Radiology and Neuroradiology, Asklepios Clinic Hamburg-Barmbek, 21033 Hamburg, Germany; m.warnke@asklepios.com (M.-M.W.); r.bruening@asklepios.com (R.B.); mar.schneider@asklepios.com (M.A.S.); 3 Biostatistics, ProResearch, Asklepios Hospital St. Georg, 20099 Hamburg, Germany; p.wohlmuth@asklepios.com; 4 Department of Gastroenterology, Hepatology and Endocrinology, Hannover Medical School, 30623 Hannover, Germany; saborowski.anna@mh-hannover.de (A.S.); vogel.arndt@mh-hannover.de (A.V.)

**Keywords:** percutaneous hepatic perfusion (PHP), melphalan, uveal melanoma

## Abstract

**Simple Summary:**

Percutaneous hepatic perfusion (PHP) with melphalan is an innovative technique that facilitates the delivery of high-dose chemotherapy to hepatic tumors while limiting systemic toxicity. Patients with uveal melanoma often initially develop hepatic metastases, which are responsive to melphalan; thus, they are particularly eligible candidates for PHP. Additionally, effective systemic therapies for metastatic uveal melanoma are still lacking; hence, further insight into liver-directed methods such as PHP is crucial. The aim of this retrospective two-center study was to pool the data of patients with liver-dominant metastatic uveal melanoma treated with PHP and analyze peri-interventional complications, response, and survival.

**Abstract:**

Percutaneous hepatic perfusion (PHP) delivers high-dose melphalan to the liver while minimizing systemic toxicity via filtration of the venous hepatic blood. This two-center study aimed to examine the safety, response to therapy, and survival of patients with hepatic-dominant metastatic uveal melanoma (UM) treated with PHP. A total of 66 patients with liver-dominant metastasized uveal melanoma, treated with 145 PHP between April 2014 and May 2020, were retrospectively analyzed with regard to adverse events (AEs; CTCAE v5.0), response (overall response rate (ORR)), and disease control rate (DCR) according to RECIST1.1, as well as progression-free and overall survival (PFS and OS). With an ORR of 59% and a DCR of 93.4%, the response was encouraging. After initial PHP, median hepatic PFS was 12.4 (confidence interval (CI) 4–18.4) months and median OS was 18.4 (CI 7–24.6) months. Hematologic toxicity was the most frequent AE (grade 3 or 4 thrombocytopenia after 24.8% of the procedures); less frequent was grade 3 or 4 hepatic toxicity (increased aspartate transaminase (AST) and alanine transaminase (ALT) after 7.6% and 6.9% of the interventions, respectively). Cardiovascular events included four cases of ischemic stroke (2.8%) and one patient with central pulmonary embolism (0.7%). In conclusion, PHP is a safe and effective salvage treatment for liver-dominant metastatic uveal melanoma. Serious AEs—though rare—demand careful patient selection.

## 1. Introduction

Melanomas of the choroid, iris, and ciliary body are collectively known as uveal melanomas (UM) [1]. In adults, UM are the most common primary intraocular malignant tumor. Manifestations of UM before adulthood are rare; the age at presentation is mostly between 50 and 70 years [2,3]. Several successful therapy options are available for the treatment of primary uveal melanoma, including enucleation, thermotherapy, or radiotherapy. Nonetheless, a vast number of patients ultimately develop metastases [4,5]. As there is no lymphatic drainage of the uvea, the tumor cells spread hematogenously and the most commonly affected organ is the liver (89%) [6].

Once patients develop liver metastases, the only potentially curative approach is surgical resection [7]. Unfortunately, less than 10% of patients that are diagnosed with metastatic disease are suitable and meet the criteria for surgery [8]: although patients with UM are comparatively young and usually present with only minor comorbidities, resectability is frequently limited by the number and anatomical location of the tumors, as the metastases are often multilocular and diffusely distributed within the liver parenchyma.

Some metastases are detected by surveillance and others after the development of symptoms [9]. Several studies have demonstrated that periodic liver imaging allows the identification of liver metastases prior to the development of symptoms [10,11]. Although a survival benefit for surveillance has not yet been proven, some centers perform the periodic screening of uveal melanoma patients, often using magnetic resonance imaging (MRI). However, there is currently no standard surveillance strategy [12]. Hence, both the intervals at which imaging should be performed and the optimal diagnostic imaging modality (MRI, computed tomography (CT), or ultrasound) remain enigmatic.

The clinical prognosis for patients with metastatic UM is dependent on tumor growth control [13]. Due to the aggressive nature of the tumor and the rather ineffective therapies, 80% of patients with liver metastases die within the first year [14,15]. To date, there is no standard first-line therapy for patients with non-resectable and multilocular liver metastases and therapy options vary between systemic therapies (e.g., systemic chemotherapy or immunotherapy) and liver-directed therapies. Current guidelines recommend thermal ablation, hepatic artery infusion, percutaneous hepatic perfusion and/or transarterial radioembolization (TARE), or chemoembolization (TACE), tailored to the number and location of the metastases [16].

A recent meta-analysis demonstrated a longer median progression-free survival (mPFS) and median overall survival (mOS) for patients who received liver-directed therapies compared to systemic therapy [17]. Comparing liver-directed therapies, a different study showed a significantly prolonged median hepatic PFS (mhPFS) and PFS after percutaneous hepatic perfusion (PHP) compared to TACE and TARE [18]. PHP is a minimally invasive procedure for primary or secondary hepatic malignancies, which delivers high doses of melphalan via the hepatic artery and minimizes systemic toxicity through filtration of the venous hepatic blood. The effectiveness of PHP was originally confirmed in a landmark phase III study in patients with cutaneous melanoma and uveal melanoma metastatic to the liver [4].

PHP is a repeatable, yet technically advanced procedure and therefore only available in selected centers. Knowledge of the common side effects of PHP, such as hematologic toxicity due to bone marrow suppression (e.g., thrombocytopenia and anemia) [19,20], and careful patient selection is required to use PHP as a feasible and safe approach for patients with non-resectable uveal melanoma liver metastases.

There is a lack of up-to-date, randomized trials in metastatic UM and most studies regarding PHP are single-center experiences with small patient cohorts. Therefore, updated data regarding the effectiveness of PHP are needed. The aim of this two-center retrospective study was to examine the safety, response, and survival after PHP in patients with hepatic-dominant metastatic uveal melanoma.

## 2. Materials and Methods

### 2.1. Study Design

The design and data management of this retrospective two-center study were approved by the Hannover Medical School ethics committee. All patients with hepatic-dominant metastatic uveal melanoma, who received PHP at two German centers (center 1: Hannover Medical School; center 2: Asklepios Clinic Hamburg-Barmbek) based on the recommendations of the local multidisciplinary tumor board between April 2014 and May 2020, were included. Requirements for PHP were sufficient renal, hematologic, and hepatic function (hemoglobin > 8 g/dL; leukocytes > 2 thsd/μL; platelets > 50 thsd/μL; bilirubin ≤ 3 × upper limit normal; serum creatinine < 1.5 mg/dL). In addition, patients were not deemed suitable for PHP if they suffered from cardiac failure (left ventricular ejection fraction less than 40%), relevant chronic restrictive or obstructive respiratory conditions or a history of intracranial lesions with a high bleeding risk, recent (<6 month) apoplex, or transient ischemic attacks.

Patient histories, including prior therapies, imaging findings, and laboratory test results, were retrospectively recorded. Procedural details, laboratory values, and clinical reports during the inpatient stay and follow-up exams were retrospectively assessed in both centers, anonymized, and submitted for collective evaluation.

### 2.2. PHP Procedure

**Figure 1:**
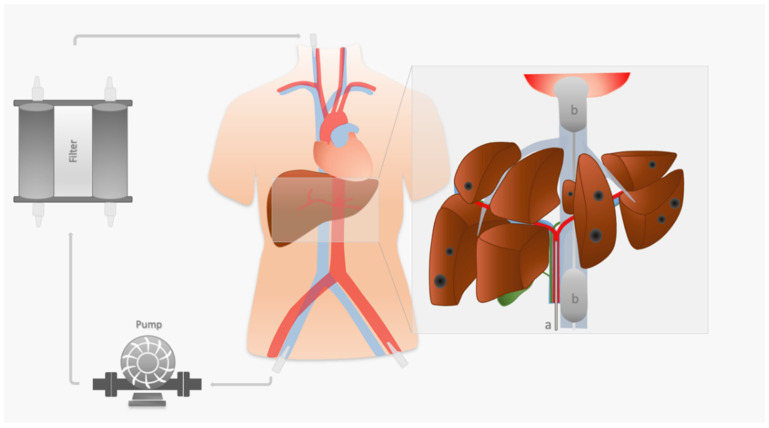
Overview of the PHP setup in a patient with inoperable disseminated hepatic metastases (dark spots on the magnified image of the liver). Introducer sheaths are inserted in the left common femoral artery and the right common femoral vein. A catheter (**a**) is placed in the proper hepatic artery to infuse melphalan in the diseased liver parenchyma (for overview reasons, we refrained from a supra-selective placement of the catheter). A double-balloon catheter (**b**) is inserted in the inferior vena cava (IVC). To isolate the hepatic segment of the IVC, the cranial balloon is inflated at the cavoatrial junction, and the caudal balloon is inflated below the confluence of the liver veins. The catheter in between the balloons is equipped with multiple side holes. Using the suction forces of a pump, the melphalan-enriched venous blood from the liver is pumped into an extracorporeal filtration system, which separates the melphalan from the blood before passing on the melphalan-cleansed blood to an introducer sheath place in the right internal jugular vein for systemic return.

In PHP, as previously described [19,20,21,22,23], a catheter, inserted through the femoral artery selectively or supra-selectively into the hepatic artery, is used for arterial chemoperfusion of the liver with melphalan (2.5–3.0 mg/kg ideal body weight up to a maximum dose of 220 mg). A double-balloon catheter, inserted via the femoral vein, isolates the hepatic segment of the inferior vena cava (IVC) and thus contains a systemic distribution of melphalan (Figure 1). The melphalan-enriched venous blood from the liver, aspirated through multiple fenestrations of the double-balloon catheter, is pumped through an extracorporeal filtration system, which separates up to 96% of melphalan [21,24]. Through a central venous catheter, the filtered blood is then returned to the systemic circulation. In the infusion phase, 500 cc of melphalan solution is infused in portions of 100 cc at a rate of 0.4 mL/s. In between the sets, an angiogram is performed to test the flow in the hepatic artery and vasodilative drugs are applied in the event of flow-restricting vasospasm. Following the infusion, the blood is filtered for an additional 30 min during the washout phase.

To maintain an activated clotting time (ACT) above 500 s, which is mandatory for safe extracorporeal hemofiltration, heparin is administered as needed, commencing with 300 U/kg body weight, followed by repeated smaller bolus injections as required.

All procedures were performed under general anesthesia due to the length of the procedure and hemodynamic changes that occur with the extracorporeal hemofiltration circuit and inferior caval vein occlusion [25]. The first day after the PHP, patients received a single shot of antibiotics as well as granulocyte colony-stimulating factor (G-CSF).

Procedural data were retrospectively collected from radiology reports and interventional protocols.

Patients were scheduled for one PHP with the option of re-treatment in case of stable disease (SD) or partial response (PR) according to the Response Evaluation Criteria In Solid Tumors 1.1 (RECIST 1.1) [26]. Contraindications for further PHP were progressive disease (PD) or unsatisfactory tolerance of the treatment.

### 2.3. Assessment of Side Effects

To categorize peri-interventional complications and toxicity, the Common Terminology Criteria for Adverse Events (CTCAE v5.0) were used. CTCAE classifies the severity of adverse events and complications according to five degrees (mild; moderate; severe; life-threatening; death). Preintervention laboratory values were regarded as baseline. Following PHP, laboratory values were determined at least after one and three days. Major adverse cardiovascular event (MACE) rate and peri-interventional mortality were assessed.

### 2.4. Assessment of Response

Follow-up exams were conducted in the respective outpatient clinics. The first follow-up imaging (CT or MRI) was performed at an average of 8.3 weeks after PHP. Response was assessed by RECIST1.1. The overall response rate (ORR) was defined as PR or complete remission (CR) and the disease control rate (DCR) as PR, CR, or SD according to RECIST1.1. Tumor mass (absolute values; cm^3^) and tumor load (relative values in relation to liver volume; %) were assessed at baseline and follow-up imaging.

### 2.5. Assessment of Survival

Median overall survival times were separately computed from the date of first PHP, from initial diagnosis of UM, and from initial diagnosis of liver involvement until latest follow-up or death, respectively. Moreover, the survival curves were compared with regard to the line of therapy.

Median PFS was calculated from the first intervention to radiological progression (according to RECIST1.1), last follow-up, or death (whichever occurred first); median hepatic PFS was concordantly calculated from first intervention to hepatic progression. Time to response (TTR) was defined as the median time from the start of treatment to the first response observed for patients who achieved a CR or PR.

### 2.6. Statistical Analysis

GraphPad Prism version 9.2.0 was used to calculate mPFS and mOS with Kaplan–Meier estimators. Comparisons between the survival curves were computed using logrank (Mantel–Cox) test. A spike histogram was applied to associate tumor mass (as continuous variables) with binary response classifications. A proportional odds model was applied to examine the impact of the line of therapy on the first response after PHP. Results were summarized by odds ratios and 95% confidence intervals (CI). Software (R Core Team 2021) was used to compute the proportional odds model. A *p*-value of <0.05 was determined to be significant.

Data of 48 patients (mostly with a shorter observational interval) were already included in single-center observational studies [20,27,28].

## 3. Results

Altogether, 66 patients with liver-dominant metastatic UM were treated with 145 PHP (median 2 (min–max: 1–6) PHP per patient) between April 2014 and May 2020 (center 1:43 patients/93 PHP; center 2:23 patients/52 PHP).

### 3.1. Patient and Interventional Data

The median age of the patients at initial diagnosis of UM was 58 (IQR 51–67) years. Five patients presented with synchronous hepatic metastasis; in the remaining patients, the median time between initial diagnosis of UM and first diagnosis of hepatic metastasis was 28 (16–60) months. In total, 48% of the patients had received other liver-directed local (38%) or systemic (18%) therapies prior to receiving PHP after a median of 6 (3–19) months after initial diagnosis of liver metastases. Moreover, 52% received PHP as first-line hepatic therapy 4 (3–8) months after the diagnosis of hepatic tumor manifestation. For details, see Table 1.

At the time of the first PHP, the median tumor load was 5% (1–12%) and the median LDH value was 284 (212–438) U/l. All PHP procedures were technically successful. Both centers used the second-generation filtration system for all PHP procedures. Table 1 and Table 2 present detailed demographic, clinical, and interventional data.

### 3.2. Toxicity

After PHP, 24.8% of patients experienced CTCAE grade 3 or 4 thrombocytopenia. Grade 3–4 anemia was observed in 11.7% and leukocytopenia in 4.1% of the patients (Table 3). Less frequently, PHP led to hepatic toxicity with grade 3–4 increased liver enzymes (increased AST or ALT after 7.6% and 6.9% of all PHP, respectively). Rarely, impairment of liver synthesis was observed (hyperbilirubinemia and hypoalbuminemia after 4.1% of the procedures), and a combined rise in AST, ALT, and bilirubin occurred only in a single patient.

### 3.3. Intra- and Postinterventional Complications

There were no AEs of grades 3 or 4 during the PHP procedures. After PHP, major thromboembolic adverse cardiovascular events occurred in five cases, leading to a MACE rate of 3.5%:

- One case of left cerebral artery occlusion; despite immediate thrombectomy, the patient remained with persistent neurological symptoms.
- One case of basilar artery thrombosis, most likely due to ne novo auricular fibrillations. Prompt thrombectomy and pharmaceutical cardioversion were performed. The same patient developed pulmonary embolism subsequent to deep vein thrombosis (treated with anticoagulation).
- Two minor strokes without sequelae.
- One case of central pulmonary embolism with good response to conservative treatment (anticoagulation).

The peri-interventional mortality was 2.1%. Despite intensive medical care, one death occurred due to sepsis three days after the first PHP. Two patients with high tumor burden (patient 1: tumor volume 73% and LDH 3370 U/L; patient 2: tumor volume 32% and LDH 3280U/L) died shortly (3 days and 12 days, respectively) after the first PHP caused by tumor lysis syndrome combined with fast tumor progression.

Further, less directive but clinically relevant complications are summarized in Table 4.

### 3.4. Response

For 61/66 (90.9%) patients, response data were available. Three patients deceased shortly after the first PHP (see Section 3.3) and two patients were lost to follow-up after discharge. After the first PHP, 26 patients showed a radiological response (PR/CR: 42.6%), 28 patients SD (45.9%), and seven patients PD (11.5%). One of these seven patients developed extrahepatic PD only. The ORR was 59% (*n* = 36). Altogether, disease stabilization was accomplished in 57 patients (DSR: 93.4%). TTR was 1.6 (IQR 1.2–3) months.

**Figure 2:**
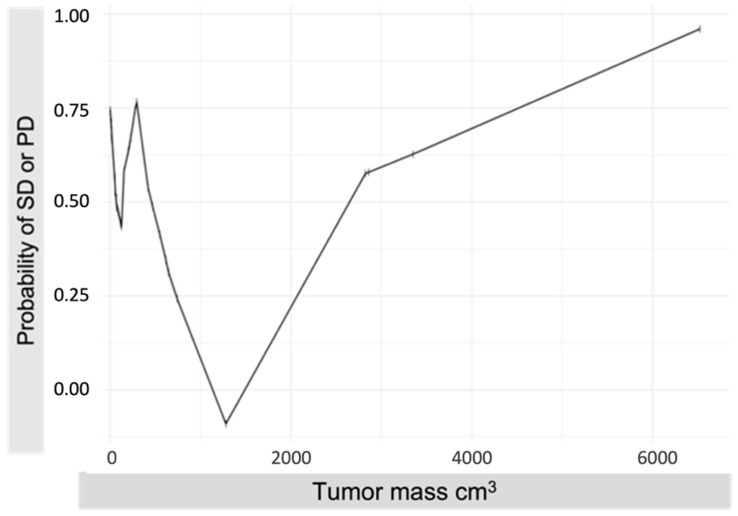
Univariable association between tumor mass and stable or progressive disease. The spike histogram shows the probability of a non-regressive response (stable disease (SD) and progressive disease (PD)) by binning the tumor mass into equal intervals and counting SD/PD in each bin. In comparison to patients with either low (<500 cm^3^) or high (>2000 cm^3^) tumor mass, patients with medium (500–2000 cm^3^) tumor mass have a lower probability of SD/PD and thus a higher probability for PR/CR.

The spike histogram (Figure 2) showed a better therapy response (PR/CR versus SD or PD) for patients with a medium tumor mass (about 500–2000 cm^3^) compared to patients with a lower or higher tumor mass.

The proportional odds model (Table 5) suggested that a higher number of therapy lines prior to PHP raises the odds of a poorer response (lines 2:1, 1.96 (1.13–3.39)).

### 3.5. Survival

**Figure 3:**
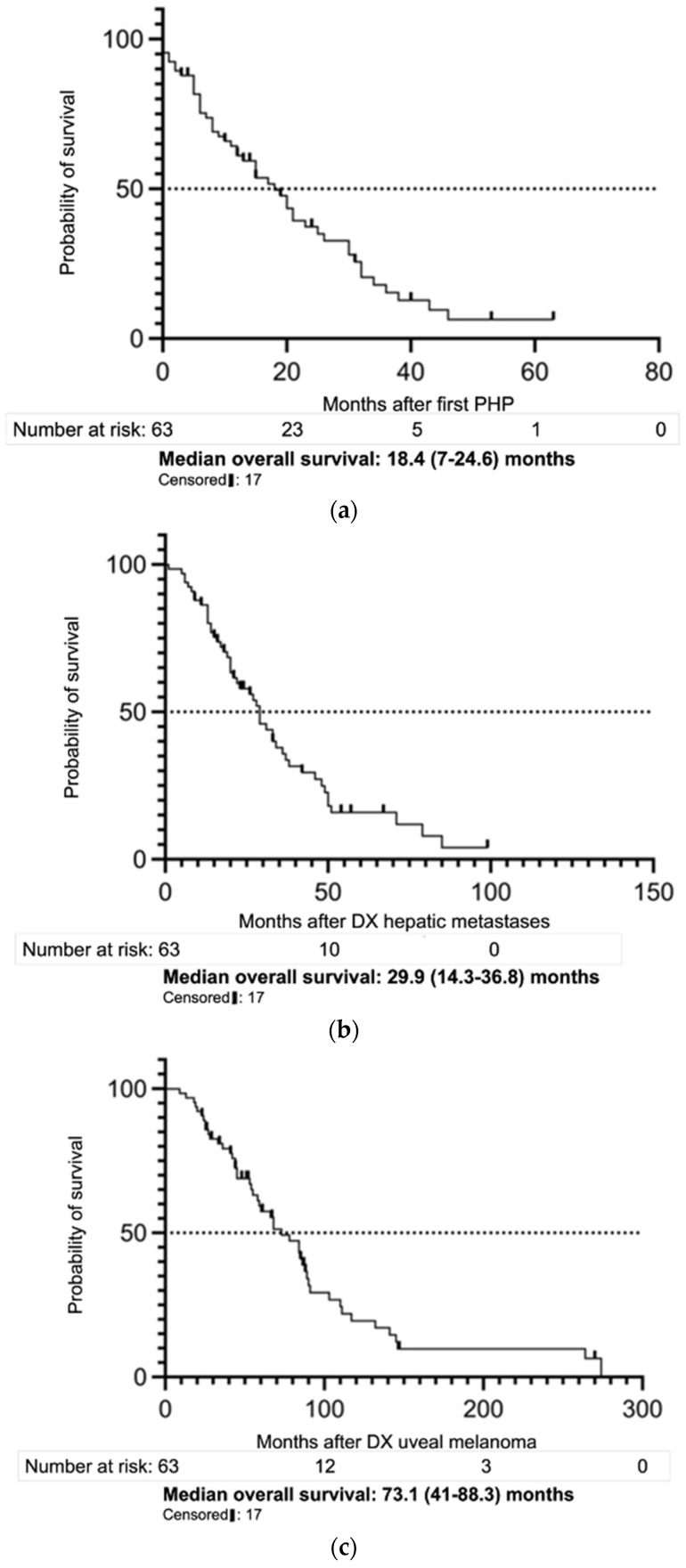
Overview of median overall survival (OS). Survival assessment after percutaneous hepatic perfusion (PHP): median overall survival (OS) in months (**a**) after the first PHP (*n* = 61), (**b**) after the date of first diagnosis (DX) of liver involvement (*n* = 61), and (**c**) after the date of DX of the uveal melanoma (*n* = 60).

Survival data of five patients were unavailable. After the time of data cut-off, median OS after first PHP was 18.4 (7–24.6) months. Calculated from the first diagnosis of hepatic metastases, median OS was 29.9 (14.3–36.8) (Figure 3). Comparisons of survival curves after first diagnosis of liver metastases showed no significant differences between patients receiving PHP as first-line therapy compared to second-to-fourth-line therapy (*p*-value: 0.16).

**Figure 4:**
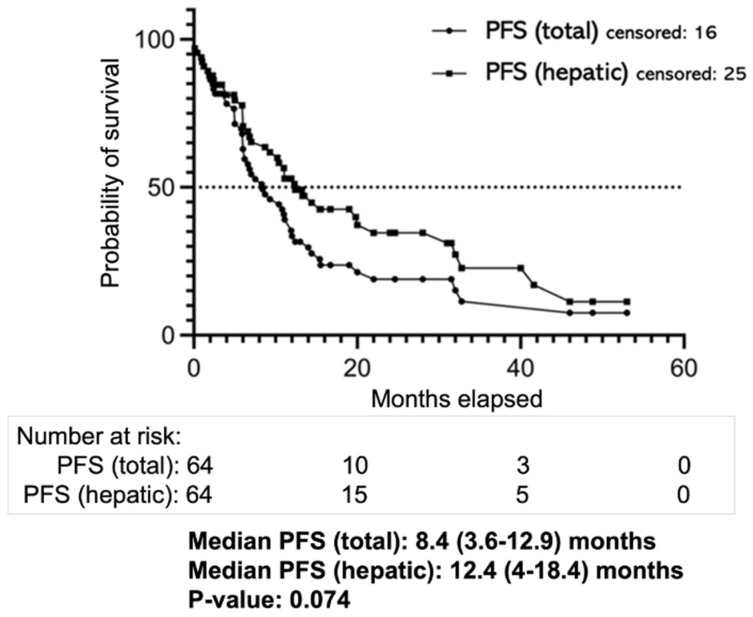
Overview of progression-free survival (PFS). Response assessment: total (overall) and hepatic progression-free survival (PFS) times since the first percutaneous hepatic perfusion (PHP).

Median PFS was 8.4 (3.6–12.9) months, whereas median hepatic PFS was 12.4 (4–18.4) months (*p*: 0.074; Figure 4).

## 4. Discussion

This retrospective analysis, which included 66 patients from two German centers who received a total of 145 PHP procedures, confirms that percutaneous hepatic perfusion (PHP) is an effective and safe method for selected patients with liver-dominant metastatic uveal melanoma (UM).

UM (which accounts for approximately 5% of all melanomas [29]) and cutaneous melanoma are very different cancers with respect to tumor biology and available treatment strategies. In UM, the primary tumor can usually be cured by surgery or radiation. However, up to half of the patients will develop metastases in the course of the disease, initially predominantly in the liver [4,8,30]. Once UM becomes metastatic, therapeutic options are limited and the survival prognosis (with a median OS of approximately one year) is dismal [31,32]. In contrast to patients with cutaneous melanoma, who benefit from the recent advances in immunotherapy and targeted therapy, metastatic UM is far less susceptible to systemic therapy options, e.g., immune checkpoint blockade [33].

With effective systemic therapies for metastatic UM still lacking [34], locoregional liver therapies can offer a promising alternative. Intraarterial approaches (such as PHP, TACE, and TARE) take advantage of the unique hepatic vascular supply: as liver metastases derive their blood solely from the hepatic artery—whereas normal liver is predominantly supplied by the portal vein—intraarterially administered drugs can bypass the non-diseased hepatic parenchyma [35]. In addition, with PHP, systemic toxicity is minimized by extracorporeal filtration.

In our study, the DCR was 93.4% and the ORR was 59%. Median PFS was 8.4 and hepatic mPFS 12.4 months, with a median OS of 18.4 months. The survival data achieved in this study are thus superior to the original phase III study for PHP (performed with the first-generation filtration system), which demonstrated enhanced tumor control (ORR: 36%, mPFS: 5.4, mhPFS: 7 months, and median OS: 10.6 months) in 93 patients treated with PHP in comparison to best alternative care (BAC) [4]. The two study arms showed no significant difference in OS, but a high crossover rate in the case of hepatic progression impaired the survival calculations. Preliminary data from an ongoing phase III trial using the second-generation filter have recently been published (FOCUS trial; Clinical trial information: NCT02678572) [36]. Whereas overall survival data are still awaited, the preliminary available data demonstrate a statistically superior ORR (32.9%) and prolonged PFS (9 months) after PHP in comparison with BAC (ORR: 13.8%; PFS: 3 months).

Our results are not only similar to these preliminary response rates but also in line with current (mostly monocentric and retrospective) studies evaluating response and survival after PHP in UM [27,28,35,37,38].

The safety of TARE with Yttrium-90 has been demonstrated in small cohorts of patients with metastatic cutaneous or uveal melanoma, with median OS rates ranging from 8 to 10 months [35,39]. Median OS with TACE in metastatic UM has been reported of up to 4–9 months [35,40]. In a retrospective comparison of survival data from 30 patients with ocular and uveal melanoma, Abbott et al. [18] found significantly prolonged hepatic PFS, overall PFS, and OS in patients treated with PHP (hPFS: 361 days; PFS: 245 days; OS: 608 days) compared to patients treated with TACE (hPFS and PFS: 52 days; OS: 265 days) or TARE (hPFS and PFS: 54 days; OS 295 days). Thus far, no controlled direct comparison between the different intraarterial treatment methods has been performed. Although there are several liver-directed therapy options available, hospitals often specialize in a specific type of procedure, especially in rare tumor types. Hence, most single-center studies report on one therapy option only. The initiation of a randomized trial that aims to directly compare different local therapies could (i) help to define which patient group benefits the most from which therapy and (ii) provide robust baseline data to measure the success of new therapies.

Despite extensive studies and a continuously growing number of available immunotherapeutic agents, studies with immunotherapies in metastatic UM are still disappointing. While pembrolizumab and nivolumab as monotherapy failed to achieve promising results (response rate 4.7%; mOS for pembrolizumab 14 months and for nivolumab 10 months) [41], a very recent phase III trial presented a preliminarily longer PFS and OS in therapy-naive patients receiving tebentafusp (PFS at 6 months 31%; OS at 1 year 73%) compared to patients receiving monotherapy with pembrolizumab, nivolumab, or dacarbazine (PFS at 6 months 19%; OS at 1 year 59%) [32]. Moreover, Hepp et al. found that combined checkpoint blockade (with ipilimumab and PD-1 inhibition) can increase the response rate and achieve a mPFS of 3 months and mOS of 16.1 months, albeit at the expense of nearly 40% severe adverse events [42]. A more recent phase II study combining nivolumab and ipilimumab as a first-line therapy reported a mOS of 12.7 months and a mPFS of 3 months [43].

In general, liver-directed therapies such as PHP should not be perceived as a direct competitor to systemic therapy. On the contrary, more research regarding the possible synergistic effects of combined therapy approaches of PHP plus immunotherapy or targeted therapy should be encouraged. This follows a general trend in interventional oncology, where head-to-head studies of systemic vs. locoregional therapies are being increasingly replaced by studies evaluating different combination therapies. For example, results of an ongoing phase II trial investigating the efficacy of PHP with ipilimumab and nivolumab in metastatic UM are forthcoming (ClinicalTrials.gov Identifier: NCT04283890).

To examine the relations between tumor mass and objective response after PHP (defined as PR/CR), we found that patients with a medium tumor mass might have an advantage over patients with a high or low tumor mass. However, these results should be perceived with caution, because (i) the number of patients with a high tumor mass in our cohort was limited and (ii) in patients with small UM metastases (e.g., low tumor mass), the response might be underestimated since smaller lesions are often difficult to assess radiologically. Size differences are harder to measure and signal intensity changes (e.g., in diffusion-weighted imaging) are more difficult to assess. Hence, patients with actual PR could be rated as SD in the radiological response evaluation. This hypothesis is supported by studies that demonstrated a negative correlation between tumor volume and OS [4,27,37,38,44] and identified a small tumor volume as an independent factor for prolonged survival [27].

Though we could not demonstrate a direct survival benefit for patients treated with PHP as first-line therapy, our analysis suggests that patients treated with PHP in an earlier line of therapy after the diagnosis of hepatic metastases are more likely to respond with regression when compared to patients receiving PHP after other locoregional or systemic therapy attempts. This observation needs to be confirmed in larger studies but should already today be considered for decision making in clinical practice.

The management of UM is subject to country-specific healthcare regulations; thus, local guidelines must be taken into consideration. Melanoma guidelines are usually developed for the management of cutaneous melanoma and might contain a few recommendations for UM as well. In light of the fundamentally different tumor biology and the paucity of treatment options, however, separate guidelines for UM are important. Examples for dedicated UM guidelines include the National Comprehensive Cancer Network (NCCN) guideline for Uveal Melanoma [45] published in 2018 and the Uveal Melanoma UK National Guidelines [10]. Such specific guidelines for UM do not only include recommendations for primary tumor care but also for standardized surveillance for liver metastasis, which, in combination with earlier treatment, might eventually improve overall survival.

Hepatotoxicity and hematotoxicity are common after PHP [35] and are related to various factors. First, although the second-generation filter system removes up to 96% of melphalan and thus greatly reduces systemic exposure, residual amounts of the chemotherapeutic agents will inevitably enter the systemic circulation. In addition, the retention of melphalan within the hepatocytes and tumor might lead to the late release of the toxic agent even after the 30 min washout phase. Furthermore, leakage of melphalan alongside the double-balloon catheter during PHP, which could expose the patient to toxic doses of melphalan, is possible—though unlikely due to accurate positioning and monitoring of the double-balloon catheter. With (transient) thrombopenia being the primary complication, our data are in line with those of available studies regarding the toxicity of PHP [19,20,27,37,46].

Both study centers experienced cardiovascular complications, including three patients with ischemic strokes (of which two were minor and one was major) and one patient with a central pulmonary embolism. Another patient suffered from a basilar artery thrombosis (most likely due to the onset of auricular fibrillations) and also developed a pulmonary embolism after suffering from a deep vein thrombosis. Concordant to our findings, occasional thromboembolic complications following PHP procedures have been reported in the literature [4,22,37].

The periinterventional mortality was 2.1%. Caused by a combination of tumor lysis syndrome and swift tumor growth, two patients died shortly after the first intervention. Both patients had a high tumor burden—in both cases, patients and medical staff were aware of the high risk and PHP was performed after extensive interdisciplinary discussions in consensus with the patients. Fulminant sepsis led to another death shortly after the first PHP.

These cases demonstrate that interdisciplinary risk assessment including the identification of potential risk factors for cardiovascular complications via cardiological and neurological assessment and close follow-up monitoring is essential, especially in patients with a high tumor burden. Apart from patient-related risk factors for cardiovascular complications, therapy-related risk factors, which, to our knowledge, have not been evaluated yet, should be looked at more closely.

A major limitation of this study is the retrospective study design, with all its potential confounders. Furthermore, due to the bicentric study design, confounding inter-center differences might be underestimated. Adverse events and complications can be influenced by various factors, such as the anesthesiologic protocol, positioning of the double-balloon catheter, and intensive care follow-up, which, though determined in a designated protocol, can be modified and individualized. Since only a few specialized centers in Europe offer PHP, most patients were referred to the treatment centers from distant locations. This might (i) lead to a possible underestimation of (long-term) complications and (ii) confound survival calculations, as the effect of possible subsequent therapies cannot be subtracted. Moreover, in patients receiving PHP not as the first therapy line, potential (residual) effects of previous therapies on survival times must be considered.

In conclusion, we demonstrate that PHP is an efficacious, minimally invasive treatment modality, which offers local tumor control in appropriately selected UM patients with primarily liver-based disease.

## Figures and Tables

**Table 1:** Patient demographics and clinical characteristics of all patients (*n* = 66) treated with percutaneous hepatic perfusion (PHP).

Parameter	Value	%
Male	30	45
Female	36	55
Age (years) ^1^	58 (51–67)	
Tumor load (%) prior to PHP ^1,2^	5 (1–12)	
Tumor mass (cm^3^) prior to PHP ^1,2^	70 (21–276)	
LDH prior to PHP ^1^ (U/L)	284 (212–438)	
Previous local liver therapy	25	38
Previous systemic therapy	12	18
PHP as 1st therapy line	34	52
PHP as 2nd therapy line	17	26
PHP as 3rd and 4th therapy line	15	23

^1^ Median and interquartile range, ^2^ *n* = 65 patients were analyzed.

**Table 2:** Characteristics of the percutaneous hepatic perfusion (PHP) procedure (*n* = 145).

Parameter	Median	IQR
Melphalan dose (mg)	182	153–207
Intervention time (min)	185	174–229
Number of PHP per patient	2	1–3

IQR: interquartile range.

**Table 3:** Hematologic, hepatic, and biliary adverse events (AEs) grade 3 and 4 (as by CTCAE v.5). Reported are the numbers/percentages of AEs after the first percutaneous hepatic perfusion (PHP) procedure and after any/all PHP calculated per procedure and also per patient.

	AEs after 1st PHP *n* = 66	AEs after Any PHP % per Patient *n* = 66	AEs after Any PHP % per PHP *n* = 145
Thrombopenia			
Grade 3	10 (15.2%)	27 (40.9%)	27 (18.6%)
Grade 4	5 (7.6%)	9 (13.6%)	9 (6.2%)
Grade 3 + 4	15 (22.7%)	36 (54.5%)	36 (24.8%)
Leucopenia			
Grade 3	0 (0%)	1 (1.5%)	1 (0.7%)
Grade 4	4 (6.1%)	5 (7.6%)	5 (3.5%)
Grade 3 + 4	4 (6.1%)	6 (9.1%)	6 (4.1%)
Anemia			
Grade 3	7 (10.6%)	17 (25.8%)	17 (11.7%)
Grade 4	0 (0%)	0 (0%)	0 (0%)
Grade 3 + 4	7 (10.6%)	17 (25.8%)	17 (11.7%)
AST increase			
Grade 3	6 (9.1%)	9 (13.6%)	9 (6.2%)
Grade 4	1 (1.5%)	2 (3%)	2 (1.4%)
Grade 3 + 4	7 (10.6%)	11 (16.7%)	11 (7.6%)
ALT increase			
Grade 3	4 (6.1%)	8 (12.1%)	8 (5.5%)
Grade 4	0 (0%)	2 (3%)	2 (1.4%)
Grade 3 + 4	4 (6.1%)	10 (15.2%)	10 (6.9%)
Hyperbilirubinemia			
Grade 3	3 (4.5%)	5 (7.6%)	5 (3.5%)
Grade 4	0 (0%)	1 (1.5%)	1 (0.7%)
Grade 3 + 4	3 (4.5%)	6 (9.1%)	6 (4.1%)
Hypoalbuminemia			
Grade 3	2 (3%)	6 (9.1%)	6 (4.1%)
Grade 4	0 (0%)	0 (0%)	0 (0%)
Grade 3 + 4	2 (3%)	6 (9.1%)	6 (4.1%)

**Table 4:** Further CTCAE grade 3–4 non-hematotoxic and non-hepatotoxic complications after 145 percutaneous hepatic perfusions (PHPs) in 66 patients.

Complication (Treatment)	*n* =	%
Active bleeding at puncture site with subsequent hemorrhagic shock (surgery)	1	0.7
Ulcer bleeding (surgical care)	1	0.7
NSTEMI (PTCA) ^1^	1	0.7
Tumor lysis syndrome	1	0.7
Acute kidney failure	1	0.7
Sepsis	1	0.7
Tachyarrhythmia absoluta	1	0.7

NSTEMI: non-ST-segment elevation myocardial infarction; PTCA percutaneous transluminal coronary angioplasty. ^1^ Immediate cardiac catheterization was performed, no abnormalities were found on coronary angiography.

**Table 5:** Proportional odds model relating line of therapy to response after therapy (CR/PR versus SD versus PD). The parameter estimates β, the standard errors S.E., the statistic (Wald Z), the *p*-value (Pr (>|Z|)), the difference between the higher and lower value of the predictor being compared (Difference), the odds ratio (Odds ratio), and the 95% confidence interval (Lower 95% CI, Upper 95% CI). PHP: percutaneous hepatic perfusion.

Variable	β	S.E.	Wald *Z*	Pr (>|*Z*|)	Difference	Odds Ratio	Lower 95% CI	Upper 95% CI
Number of therapy lines prior to PHP	0.6707	0.2799	2.40	0.0166	1	1.956	1.130	3.385

## Data Availability

The data presented in this study are available on request from the corresponding author. The data are not publicly available due to privacy.
